# Nitrogen-Doped Carbon Aerogels Derived from Starch Biomass with Improved Electrochemical Properties for Li-Ion Batteries

**DOI:** 10.3390/ijms22189918

**Published:** 2021-09-14

**Authors:** Marcelina Kubicka, Monika Bakierska, Krystian Chudzik, Michał Świętosławski, Marcin Molenda

**Affiliations:** Faculty of Chemistry, Jagiellonian University, Gronostajowa 2, 30-387 Krakow, Poland; marcelina.lis@doctoral.uj.edu.pl (M.K.); krystian.chudzik@doctoral.uj.edu.pl (K.C.); m.swietoslawski@uj.edu.pl (M.Ś.)

**Keywords:** Li-ion battery, anode material, carbon aerogel, biomass, starch, N-doping, chemical modification, functionalization

## Abstract

Among all advanced anode materials, graphite is regarded as leading and still-unrivaled. However, in the modern world, graphite-based anodes cannot fully satisfy the customers because of its insufficient value of specific capacity. Other limitations are being nonrenewable, restricted natural graphite resources, or harsh conditions required for artificial graphite production. All things considered, many efforts have been made in the investigation of novel carbonaceous materials with desired properties produced from natural, renewable resources via facile, low-cost, and environmentally friendly methods. In this work, we obtained N-doped, starch-based carbon aerogels using melamine and N_2_ pyrolysis as the source of nitrogen. The materials were characterized by X-ray powder diffraction, elemental analysis, X-ray photoelectron spectroscopy, galvanostatic charge–discharge tests, cyclic voltammetry, and electrochemical impedance spectroscopy. Depending on the doping method and the nitrogen amount, synthesized samples achieved different electrochemical behavior. N-doped, bioderived carbons exhibit far better electrochemical properties in comparison with pristine ones. Materials with the optimal amount of nitrogen (such as MCAGPS-N8.0%—carbon aerogel made from potato starch modified with melamine and CAGPS-N1.2%—carbon aerogel made from potato starch modified by N_2_ pyrolysis) are also competitive to graphite, especially for high-performance battery applications. N-doping can enhance the efficiency of Li-ion cells mostly by inducing more defects in the carbon matrix, improving the binding ability of Li^+^ and charge-transfer process.

## 1. Introduction

Demand for naturally sourced and artificial graphite, which is widely used as anode material in lithium-ion batteries (being the second-largest component by weight), is projected to increase in the next years [1,2]. The major factor driving the growth of the graphite market is the surge of global cell production happening on the back of the growth in the worldwide demand for electric vehicles and renewable energy storage systems. In this context, an important motivation to undertake further research in terms of carbonaceous anode materials is the desire for a withdrawal from the use of finite or synthetic (often toxic or expensive) carbon sources. Thus, employing sustainable reserves is more reasonable with regard to securing a supply chain, price stability, and compliance with stringent environmental regulations [3,4,5]. Many different types of natural sources, such as starch [6,7,8], cellulose [9], lignin [10], corn products [11,12], palm spathe [13], bamboo chopsticks [14], coffee grounds [15], and mushrooms [16], have been employed so far to prepare carbon anode materials with distinctive performance, which makes biopolymer precursors promising resources for the preparation of carbon material with a view to apply them in lithium-ion batteries. Our previous research demonstrated that the use of starch is particularly interesting [17,18,19,20]. The idea of such a concept—apart from the fact that starch is a widely available, renewable, and affordable raw material that forms products of green technology with zero carbon footprint—has its justification in the wide diversity of this polymer, depending on the botanical origin (concerning a different proportion of amylose and amylopectin as well as the different length of the polymer chain in various types of starch). Hence, the properties of resulting starch-based carbon aerogels (CAG materials) can be easily affected and controlled by the precursor composition, allowing for the design of materials with strictly defined properties depending on the requirements of their application [17,18,19]. Another important issue is that the properties of CAGs not only depend on the type of starch used in their production, but also on the method of synthesis and conditions of the thermal treatment process [20]. It is also worth noticing that carbon aerogels derived from the starch precursor are obtained through ecofriendly (water-based) and facile synthesis (not demanding high-pressure, complex, multistep treatment or very high temperatures during the carbonization process) [17]. Furthermore, the properties of CAG materials can also be easily customized by applying appropriate chemical modifications [21]. Nevertheless, recently, more and more reports on the subject of carbonaceous materials functionalization relate to actions that are connected with introducing various types of functional groups into the structure and/or onto the surface of carbon materials that significantly change their chemical nature [22,23,24,25,26]. One of the most interesting and effective approaches is nitrogen incorporation. Nitrogen-doped carbons exhibit unique physicochemical and electrochemical properties [27,28,29,30,31,32] that are useful in a wide range of practical application [33]. In general, most carbon precursors have very low nitrogen content. For this reason, the amount of nitrogen functional groups on the surface of unmodified carbons is generally low. Most of the methods for enriching carbon with nitrogen can be attributed to one of two main synthetic routes: thermal treatment of precursor or carbon compound in the presence of a nitriding agent as well as impregnation of the carbons with nitrogen-containing agent [32,34,35,36,37,38], or carbonization of plastics containing nitrogen in their structure [39,40]. Depending on the modification conditions and the applied methods, the type of nitriding agent, and the stage of the technological process of carbon production on which the modification was carried out, modified carbonaceous materials show various physical, chemical, electronic, and electrochemical properties.

In this work, to promote the electrochemical performance of carbon nanostructures derived from starch precursor and to further increase their utility in high-performance lithium-ion batteries, we report a comprehensive study on the preparation and characterization of modified analogs via incorporation of nitrogen functional groups into the structure of carbonaceous materials.

## 2. Results

Elemental analysis results of obtained carbon aerogels are given in Table 1. For all samples, “others” means oxygen, which is always available in the carbon material. From the data, the CAGPS sample has a higher carbon content (~94%) than nitrogen-doped samples (much higher than the samples modified with the use of melamine, up to ~15%; slightly higher than the sample pyrolyzed in nitrogen atmosphere, by ~1.5%). Moreover, the original carbon aerogel has the lowest oxygen content (~4.8%), though it is comparable to the CAGPS-N1.2% sample (~4.9%). Importantly, the mass percentage of nitrogen in the products increases from 0.3% to almost 8% upon addition of melamine from 1wt. % to 20 wt. % during the synthesis process. The nitrogen content in the sample unmodified with melamine but pyrolyzed in N_2_ atmosphere (CAGPS-N) was estimated to be ~1.2%. The highest nitrogen (~8%) and interestingly, at the same time, oxygen content (~11%) was observed in the MCAGPS-N8.0% sample. As for the amount of hydrogen, it varies slightly between samples and ranges from 1.2% to about 1.9%. Our analysis indicates that both the chemical and physical modification of starch-based aerogels leads to their successful functionalization and the introduction of nitrogen to the structure of carbonaceous materials.

**Figure 1:**
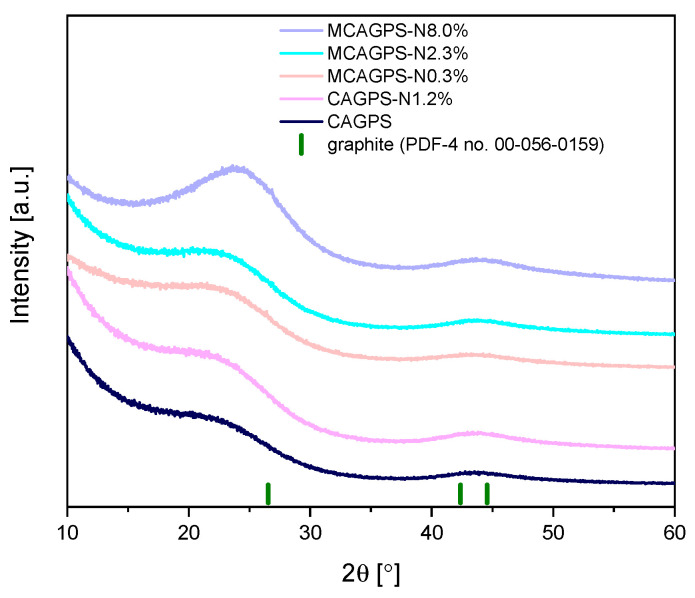
XRD patterns of nitrogen-doped and pristine carbon aerogels over the 2θ range of 10–60° (MCAGPS—carbon aerogel made from potato starch modified with melamine; CAGPS-N—carbon aerogel made from potato starch modified by N_2_ pyrolysis; CAGPS—pristine carbon aerogel).

The XRD patterns of nitrogen-doped carbon aerogels (MCAGPS-N and CAGPS-N samples) and reference nitrogen-free carbon (CAGPS sample) are shown in Figure 1. These patterns exhibit similar diffraction features with two broad, low-intensity peaks at approximately 2θ = 24° and 44°, corresponding to the (002) and (101) crystal planes, respectively, that are typical of amorphous, disordered carbon materials with a low degree of graphitization. No other impurities were observed in the XRD patterns. As MCAGPS-N and CAGPS-N samples have similar diffraction peaks to the pristine carbon (CAGPS), it can be concluded that nitrogen-doping does not cause significant variation in the carbon crystal structure. Nevertheless, with an increase in nitrogen content, the peaks visible in Figure 1 become more prominent and narrow, indicating the improvement of the graphitization degree. This is also reflected in the values of the interlayer distance of the (002) plane (d_002_) calculated by Bragg’s law, which is 0.3875 nm for the CAGPS and 0.3675 nm for the MCAGPS-N8.0% sample (d_002_ for graphite is 0.3355 nm). Interestingly, the larger d_002_ values of the MCAGPS-N and CAGPS-N materials (which, for all obtained samples, is between 0.3675 and 0.3845 nm) in comparison with that of graphite implies that the intercalation of nitrogen results in an enlarged interlayer distance of porous carbon, which can potentially facilitate the diffusion and insertion/extraction of Li^+^ in the carbon matrix.

**Figure 2:**
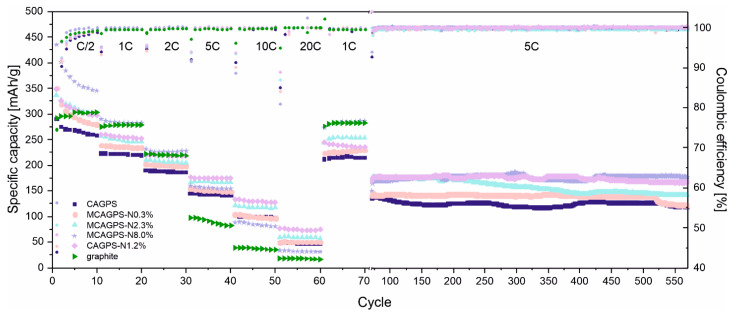
Galvanostatic charge–discharge tests of investigated samples in half-cells under different current loads (MCAGPS—carbon aerogel made from potato starch modified with melamine; CAGPS-N—carbon aerogel made from potato starch modified by N_2_ pyrolysis; CAGPS—pristine carbon aerogel).

**Figure 3:**
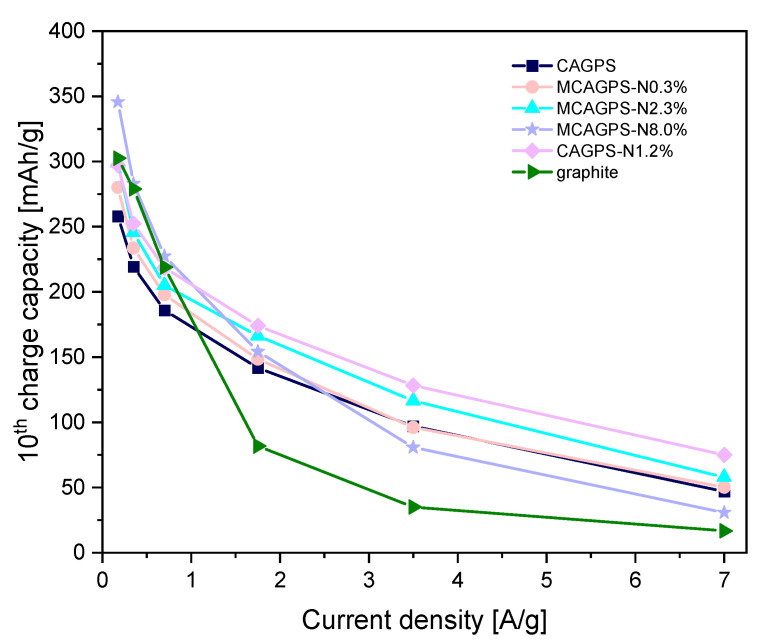
Charge capacity of every 10th cycle as a function of current density (MCAGPS—carbon aerogel made from potato starch modified with melamine; CAGPS-N—carbon aerogel made from potato starch modified by N_2_ pyrolysis; CAGPS—pristine carbon aerogel).

The nitrogen-doped carbon aerogel materials, as well as the pristine ones (CAGPS), were subjected to galvanostatic charge–discharge tests in a half-cell system. All studied carbons were tested at room temperature (Figure 2 and Figure 3), performing 7 sets of 10 cycles in sequence using currents equivalent to C/2, 1C (0.35 A/g), 2C, 5C, 10C, 20C, 1C (in reference to graphite); then, an additional 500 cycles under 5C current rate were performed (excluding graphite due to its bad performance under higher current load).

For all CAG cells, low values of initial Coulombic efficiency (CE) are observed. This capacity loss is typical for starch-based carbon aerogel materials [17,18,19,20,21] and can be attributed to the side reactions, such as SEI film formation at the electrode–electrolyte interface or the irreversible reaction of Li^+^ with residual oxygenated functional groups at these carbon surfaces. However, with the increase of nitrogen in the investigated samples, a slight growth of the initial CE (connected with smaller irreversibility of lithium-ion consumption) can be noticed—from 43.95% for CAGPS to 45.52%, 48.40%, 50.17%, and 56.43% for MCAGPS-N0.3%, CAGPS-N1.2%, MCAGPS-N2.3%, and MCAGPS-N8.0%, respectively. During the first 10 cycles for tested aerogels, the specific capacities suffer from the quick drop, which is related to the electrode–electrolyte activation process. Then, in the next cycles, together with the formation of an SEI layer, a considerable growth in efficiency is observed. After complete growth of the stable SEI, the actual stability of the materials and the proper reversibility of Li^+^ insertion–extraction reactions are reached, indicating a good cycling performance. Going further, for lower current rates (up to 2C), the charge capacity of analyzed materials increases in the following order: CAGPS < MCAGPS-N0.3% < MCAGPS-N2.3% < CAGPS-N1.2% < graphite < MCAGPS-N8.0% (Figure 3). For N-doped samples with melamine as a source of nitrogen, a higher amount of nitrogen is introduced and a higher specific capacity is obtained. On the other hand, under elevated current loads, the highest capacity is achieved by CAGPS-N1.2% and the worst by unmodified graphite. At current rates, 5C and 10C CAGPS-N1.2% might be reversibly charged to 174.0 mAh/g and 128.3 mAh/g while the corresponding values of graphite are only 81.8 mAh/g and 35.1 mAh/g. For the CAGPS-N1.2%, the capacity remains at 75 mAg/h even with 20C (7.0 A/g) and, when the current is reduced to 1C, the capacity quickly returns to 235 mAh/g, proving excellent charge transfer kinetics (which is also observed for the remaining examined carbons). The cycle performance was also examined with long-term galvanostatic charge–discharge tests. It can be noticed that cells with CAGPS-N1.2% and MCAGPS-N8.0% as electrode materials are characterized by the most efficient operating parameters. 

After 500 cycles of working under 5C current load, they could maintain the charge capacities of 166.4 mAh/g (for CAGPS-N1.2%) and 177.6 mAh/g (for MCAGPS-N8.0%) with capacity retention of 98.2% and 117.4%, respectively. The unexpected growth of the charge capacity with cycling for MCAGPS-N8.0% might be ascribed to the activation process under higher current rates (the unexposed pores become more accessible after repeated testing). Hence, N-doped carbon aerogels can be ascribed as electrochemically stable and efficient alternatives for graphite, especially for high-performance Li-ion applications.

**Figure 4:**
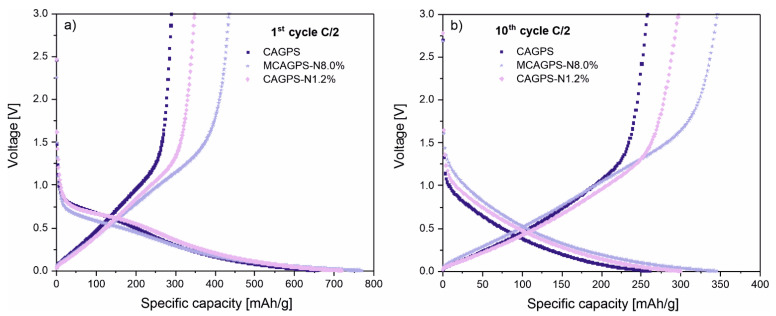
Charge–discharge voltage profiles for the (**a**) 1st and (**b**) 10th cycle of CAGPS-, CAGPS-N1.2%-, and MCAGPS-N8.0%-based electrodes at C/2 current rate (MCAGPS—carbon aerogel made from potato starch modified with melamine; CAGPS-N—carbon aerogel made from potato starch modified by N_2_ pyrolysis; CAGPS—pristine carbon aerogel).

Figure 4 compares the shape of potential curves for the most effective working cells CAGPS-N1.2% and MCAGPS-N8.0% with reference to CAGPS for the 1st and 10th cycles at the C/2 current rate. The presented voltage profiles for N-doped materials are quite similar to the pristine ones. However, the N-species developed on the surface may slightly change the exact conditions of the Li^+^ insertion as well as SEI layer formation. Besides, there is an obvious difference in the exact voltage values for the initial and following cycles. For the 1st cycle, two plateaus can be distinguished—the sloping one at about ~0.8 V (common for all carbon-based anodes) is due to the formation of SEI layer on the surface of carbon electrodes, and the plateau at ~0.25 V (detected in all profiles) corresponds to the intercalation of lithium ions in starch-based carbon aerogel materials. For the 10th cycle, all curves can also be divided into two regions—a pure slope above ~0.25 V and the mentioned plateau starting at about 0.25 V.

**Figure 5:**
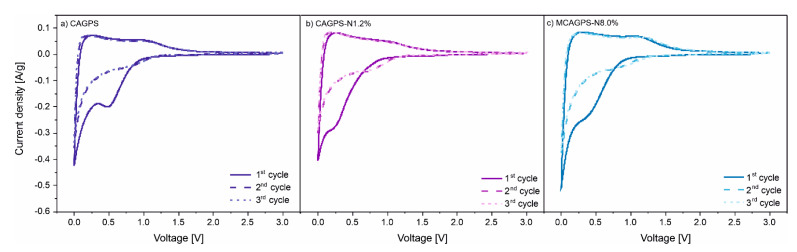
Cyclic voltammetry curves of (**a**) CAGPS-, (**b**) CAGPS-N1.2%-, and (**c**) MCAGPS-N8.0%-based cells (MCAGPS—carbon aerogel made from potato starch modified with melamine; CAGPS-N—carbon aerogel made from potato starch modified by N_2_ pyrolysis; CAGPS—pristine carbon aerogel).

Cyclic voltammetry curves (measured at a scan rate of 0.1 mV/s) of CAGPS, CAGPS-N1.2%, and MCAGPS-N8.0% are presented in Figure 5. Voltammograms of all samples show a broad cathodic peak, visible only during the first cycle, which corresponds to the formation of a passivation layer on the anode materials surface. Analyzed samples present a significant change in the potential of SEI layer formation, assuming values of 0.47 V, 0.26 V, and 0.17 V for CAGPS, MCAGPS-N8.0%, and CAGPS-N1.2%, respectively. This implies a different structure of the SEI layer and can also be a reason for differences in performance during galvanostatic cycling. As is commonly known for graphite, the first cycle of lithiation may be divided into several continuous transitions (from LiC_72_ to LiC_6_), during which the voltage of distinct reaction between delithiated and fully lithiated carbon varies in the range of 0.01 V to 1.50 V. The irreversible capacity of first discharge above 0.25 V is attributed to the reactions, which take place before the beginning of intercalation. Reactions occurring between 0.25 V and 0.04 V are connected with the formation of a solid electrolyte interface during the lithiation of graphite [41]. Voltammetry measurements show that the passive layer formation of reference carbon (CAGPS) takes place at higher potentials. After introducing nitrogen into the structure, doped material seem to be less sensitive to side reactions before the first lithiation. As mentioned above, the slight increase of coulombic efficiency in the initial cycle after doping (43.95% for CAGPS, 48.40% for CAGPS-N1.2%, and 56.43% for MCAGPS-N8.0%) can be connected with the partial suppression of the side reactions of the material on the contact surface with the liquid electrolyte.

**Figure 6:**
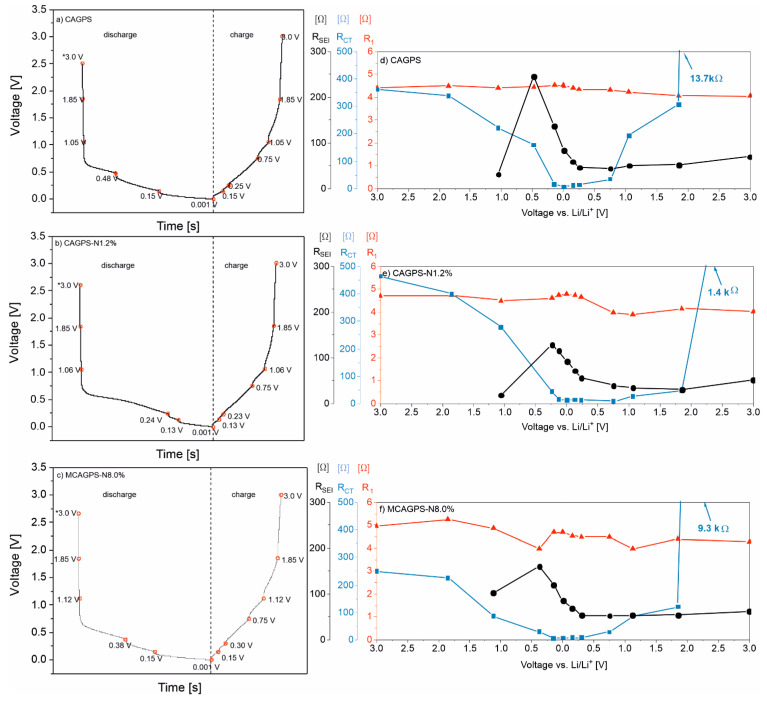
Voltage profiles of (**a**) CAGPS, (**b**) CAGPS-N1.2%, and (**c**) MCAGPS-N8.0% materials with marked potentials, in which electrochemical impedance spectroscopy (EIS) measurements were carried out. The values of R_1_, R_SEI_, and R_CT_ as functions of potential for (**d**) CAGPS, (**e**) CAGPS-N1.2%, and (**f**) MCAGPS-N8.0%, respectively ((MCAGPS—carbon aerogel made from potato starch modified with melamine; CAGPS-N—carbon aerogel made from potato starch modified by N_2_ pyrolysis; CAGPS—pristine carbon aerogel).

CAGPS, CAGPS-N1.2%, and MCAGPS-N8.0% samples were investigated using electrochemical impedance spectroscopy at different state-of-charge of half-cells (Figure 6). EIS spectra were simulated using R_1_(R_SEI_Q)(R_CT_Q)Q models, excluding the two first points for each material due to the absence of SEI layer before the first discharge process begins. For simulations of these points, model R_1_(R_CT_Q)Q was applied (a more detailed description can be found in our previous works [19]). R_1_ is connected to the ohmic resistance of the cell, which does not change significantly during electrochemical reactions in the half-cell. R_SEI_ represents the resistance of solid electrolyte interphase on the anode surface. A distinct rise in this resistance component is shown at the beginning of the first intercalation reaction in all three samples, although N-doped materials show lower R_SEI_ values at this point in comparison with pure CAGPS (CAGPS—245 Ω, CAGPS-N1.2%—128 Ω, MCAGPS-N8.0%—161 Ω). This may correspond to lowering the thickness of the electrolyte interphase layer and/or its better Li^+^ permeability. Fully discharged cells (0.001 V) exhibit smaller values of R_SEI_ resistances (CAGPS—84 Ω, CAGPS-N1.2%—92 Ω, MCAGPS-N8.0%—85 Ω). At full charge (3.0 V), R_SEI_ values differ greater in favor of N-doped samples (CAGPS—70 Ω, CAGPS-N1.2%—52 Ω, MCAGPS-N8.0%—62 Ω), which indicates overall stabilization of the formed SEI layer on N-doped samples. The last considered resistance R_CT_ represents the charge-transfer process in the electrode. The results of simulations show a significant decrease in this component for melamine-modified CAG (MCAGPS-N8.0%) through the whole intercalation voltage region (around 1 V–0.001 V). This is a reflection of the improved conductivity due to the introduction of nitrogen into the carbonaceous material structure. The R_CT_ decrease is also observed for N_2_-treated CAG (CAGPS-N1.2%).

**Figure 7:**
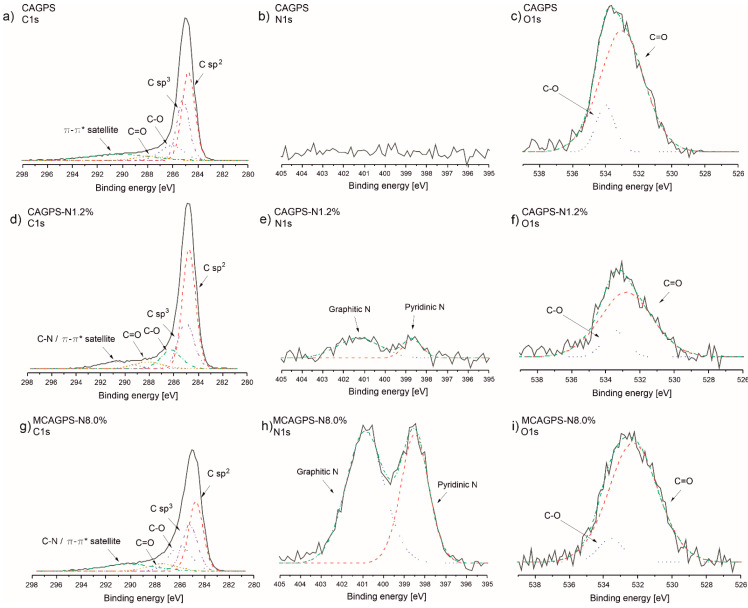
The X-ray photoelectron spectra of investigated (**a**–**c**) CAGPS, (**d**–**f**) CAGPS-N1.2%, and (**g**–**i**) MCAGPS-N8.0% materials (MCAGPS—carbon aerogel made from potato starch modified with melamine; CAGPS-N—carbon aerogel made from potato starch modified by N_2_ pyrolysis; CAGPS—pristine carbon aerogel).

In order to analyze the surface chemical composition and explain the electrochemical behavior of modified carbon aerogels, the XPS measurements for the most effective materials MCAGPS-N8.0% and CAGPS-N1.2% as well as CAGPS as reference were conducted. As depicted in Figure 7, there are recorded C1s, O1s, and N1s XPS spectra of obtained carbon aerogels. For all characterized samples, the XPS spectrum of the C1s (Figure 7a,d,g) consists mainly of the large peak at 284.8 ± 0.1 eV typical for sp^2^ graphitic carbon bonding, which indicates that most of the carbon atoms in those structures are arranged in a conjugated honeycomb lattice. Among the rest, there are observed peaks at 285.2 ± 0.2 eV, 286.1 ± 0.1 eV, 287.8 ± 0.4 eV, and a broad one at around 290–291 eV ascribed to C-C sp^3^, C-O, C=O, as well as π-π* satellite from the graphite like domain, respectively. Further, as suggested in the literature for N-doped carbons, the sp^2^ C-N may overlap with C-O peaks; however, for investigated samples, it is hard to separate them [42,43]. Figure 7b,e,h reveal the presence of O1s spectra. We noticed a combination of two characteristic peaks, among which the peak at 532.8 ± 0.5 eV is related to oxygen in the carbonyl group (C=O) and the peak at 533.5 ± 0.5 eV is assigned to phenol/ether bonding (C-O). Thus, at this point, the larger differences in the surfaces of the carbons can be seen for N1s spectra (Figure 7c,f,i). The outcomes confirm that nitrogen was successfully doped into MCAGPS-N8.0% and CAGPS-N1.2% structures. Their N1s spectra can be deconvoluted into two peaks ascribed to the N-6 (398.5 ± 0.2 eV) and N-Q (400.9 ± 0.3 eV) nitrogen species [42,44,45,46,47]. The N-6 is the pyridinic nitrogen, where one nitrogen atom is bonded with two carbon atoms in a six-membered ring and it is usually located at the edge of graphene layers. N-Q represents the quaternary (graphitic) nitrogen, which is bonded to three carbon atoms and exists in the middle of plain graphene layer position [45,47]. For both modified samples, the pyridinic is the dominant component. The concentration ratio N-6 to N-Q (calculated using the corresponding peak areas) is equal to 71.4%, and 41.3% for MCAGPS-N8.0% and CAGPS-N1.2%, respectively. Hence, the relative ratio of N-6 increases with the growth of the total amount of nitrogen in the carbon matrix. It is also worth noting that these nitrogen species have slightly different functions in their carbon structure. N-Q particularly affects the increase in the electronic conductivity of carbonaceous samples (by generating an excess of electrons). On the other hand, N-6 provides more open channels (nanopores/active sites) and, by doing so, facilitates lithium-ions’ storage and improves their fast transportation [27,48]. The relative atomic percentage of C1s and O1s calculated from XPS is equal to 88.5% and 4.3% for MCAGPS-N8.0%, 97.1% and 2.0% for CAGPS-N1.2%, and 95.4% and 4.6% for pristine carbon aerogel. The results also confirm nitrogen doping: 7.2 at% in MCAGPS-N8.0% and 0.9 at% in CAGPS-N1.2%, which coincides (within the error range) with the outcomes of the elemental analysis. Based on the XPS, it is concluded that for N-doped materials, there are higher amounts of N- and O-containing surface functional groups (in total) compared with the pristine one. All these heteroatoms may play a crucial role in Li-ion cell performance due to their higher electronegativity (causing changes in the local electron density) that gives additional benefits for lithium kinetics and storage capabilities.

## 3. Discussion

All presented results show a significant impact of N-doping on the electrochemical properties of the carbon aerogel derived from potato starch. By doping nitrogen (from different N-configurations—especially the pyridinic one) into the carbon structure, an additional number of defects (efficient active sites) are created, which further enhance Li intercalation properties. Furthermore, N-doping promotes electronic conductivity and improves kinetics performance of the electrode as well as charge-transfer transport and the binding ability of Li^+^. All these effects are connected with differences in electronegativity between carbon (X_C_ = 2.55), as well as nitrogen (X_N_ = 3.04). Higher electronegativity strengthens the electron density among N atoms, which translates into capturing greater amounts of Li^+^ (by exhibiting more favorable binding of N-doped sites with lithium ions), resulting in increasing lithium storage capacity. Further, the generation of excess electrons enhances the electronic conductivity of carbonaceous materials (N incorporated into the C-matrix in a certain sense causes n-type doping effects). N-doped carbon aerogel materials contain more oxygen than undoped ones and these oxygen surface groups can also give additional, beneficial effects and contribute to the capacity growth (X_O_ = 3.44). Moreover, our results suggest that apart from the type of N-dopant and the amount of nitrogen, the crucial for energy storage usability is a surface effect induced by N-doping. All these aspects must be considered to fully explain the improvement of N-doped aerogel materials. Most suitable for high-capacity lithium storage (also confirmed by earlier research [49,50]) is pyridinic carbon, which is responsible for the enhancement of Li storage and more helpful for increasing reversible capacity than other types of N-dopants (like N-5, NQ) [45,51].

## 4. Materials and Methods

### 4.1. Synthesis of Carbon Aerogel Derived from Potato Starch (CAGPS) and N-Doped Carbon Aerogel Materials (MCAGPS-N and CAGPS-N)

To obtain carbon aerogel (CAGPS sample) and nitrogen-doped carbon materials (MCAGPS-N and CAGPS-N samples), proper compositions of polymeric carbohydrate (potato starch, Sigma-Aldrich, Saint Louis, MO, USA) were, in short, processed by gelatinization route followed by the pyrolysis of organic precursors under an ambient atmosphere (Ar for CAGPS and MCAGPS-N samples and N_2_ for CAGPS-N sample) at 700 °C, according to the method reported in our previous studies [17,18,19,20,21]. Importantly, and applicable to MCAGPS-N samples, is the fact that the main precursor of carbon materials–potato starch, was modified with the addition of varied melamine (Acros Organics, Belgium, 99%) concentrations (1%, 5%, and 20 wt. %). In this case, potato starch with melamine was homogeneously dispersed in distilled water and, as for all other samples, the dispersions were continuously stirred at 80 °C in an oil bath until gelatinization occurred. The obtained sols were aged for 1 day and the samples were poured over with 96% ethanol solution (from Borzęcin, Poland) and left tightly sealed. After 5 days since the exchange of the alcohol solution, the obtained alcogels were dried for 24 h at 50 °C under atmospheric pressure and, after that, subjected to pyrolysis in a tube furnace at a temperature of 700 °C under an ambient atmosphere for 6 h with a ramping rate of 2 °C/min.

### 4.2. Materials Characterization

Determination of CHN (carbon, hydrogen, nitrogen) elements content in the produced reference and modified carbons was carried out by a microanalyzer Vario MICRO cube coupled with microbalance (Elementar, Frankfurt, Germany). The structure of the carbon materials was determined utilizing X-ray powder diffraction (XRD) with a Bruker D2 PHASER diffractometer using Cu Kα1 radiation (λ = 0.154184 nm) over a 2θ range of 10–60° at a step rate of 0.02°. X-ray photoelectron spectroscopy (XPS) analysis of the aerogel samples was performed using a Prevac photoelectron spectrometer (Rogowo, Poland) equipped with a hemispherical VGSCIENTAR3000 analyzer (Uppsala, Sweden). XPS experiments were taken with mono Al-K_α_ (E = 1486.6 eV) radiation and a low-energy electron flood gun FS40APS (Prevac, Rogowo, Poland) to compensate charge on the surface during measurements. Binding energy of the carbon (284.8 eV) was used for calibration. Fitting of the XPS spectra was provided through the CasaXPS software.

### 4.3. Electrochemical Characterization

The electrochemical characterization of Li-ion half-cells based on the fabricated carbon electrodes was conducted using the galvanostatic charge–discharge tests (GCDT). Additionally, for the most distinctive samples, galvanostatic cycling accompanied by electrochemical impedance spectroscopy (GC+EIS) and cyclic voltammetry (CV) were performed. All electrochemical measurements were carried out using R2032 cointype cells. The Li/Li+/(CAG electrode) cells were assembled in an argon-filled glove box (MBraun Unilab) with both H_2_O and O_2_ levels of less than 0.1 ppm. The CAG electrodes were fabricated by mixing the 90 wt. % of active material with 10 wt. % of polyvinylidene fluoride (PVDF) binder (Sigma-Aldrich) in N-methyl-2-pyrrolidone (NMP) solvent (Sigma-Aldrich, ≤99.5%). The prepared slurry was homogenized and stirred in Planetary Micro Mill PULVERISETTE 7 premium line (Fritsch, Idar-Oberstein, Germany) for a few minutes and then coated on a copper foil. Finally, after drying at 90 °C for 24 h, the foil was cut into circular discs to form working electrodes of 12 mm in diameter. The typical loading of active materials in the assembled cells was around 1.21 ± 0.10 mg/cm^2^. As a reference electrode, a metallic lithium foil (Sigma-Aldrich, 99.9%) was used. Both electrodes were separated by a microporous trilaminate of polypropylene/polyethylene/polypropylene Celgard 2325 film (Celgard LLC) and two porous glass microfiber filters (Whatman GF/F, Sigma-Aldrich). The electrolyte was a 1 M solution of lithium hexafluorophosphate (LiPF_6_) in a mixture of ethylene carbonate (EC) and diethyl carbonate (DEC) at a volume ratio of 1:1 (Sigma-Aldrich, battery grade). The galvanostatic charge and discharge tests (GCDT) were run on the NEWARE multichannel testing system at room temperature under different current loads (from C/2 to 20C). Cut-off voltages were 0.001 V and 3.0 V for the discharge and charge processes, respectively. Galvanostatic cycling accompanied by electrochemical impedance spectroscopy (GC+EIS) as well as cyclic voltammetry were conducted on a potentiostat/galvanostat AUTOLAB PGSTAT302N/FRA2 (Metrohm Autolab, Utrecht, Netherlands). The EIS measurements were performed with different state-of-charge (SOC) of the cells by applying an alternating current signal of 0.01 V amplitude, in the frequency range from 100 kHz to 0.01 Hz, every time after 30 min relaxation in a set potential. The impedance data were fitted using Nova 1.11 Autolab software based on the Boukamp model. CV measurements, in turn, started from an open circuit voltage (OCV) with a 0.1 mV/s scan rate, in the voltage range from 0.001 V to 3 V.

## 5. Conclusions

Adding melamine at the stage of gelatinization allows obtaining starch–melamine-based xerogel. During the pyrolysis process of melamine-modified xerogels in argon flow, through the evolution of gases (CO_2_, H_2_O, and CO [18]), some cracks and holes are generated [19] that allow obtaining a unique aerogel framework. Oxygen groups are partially removed from CAG materials at high temperature and, as a result, active sites for nitrogen doping into C-matrix are formed. At the same time, during pyrolysis, by decomposition of melamine, the nitrogen species (mainly N-6) are produced. These species can attack the active sites in the carbon matrix and form nitrogen-doped sheets. On the other hand, the high-temperature pyrolysis of xerogels in N_2_ atmosphere leads to the partial removal of oxygen groups (through gases evolution) and results in the formation of active sites for nitrogen doping into C-matrix. At the same time, gaseous nitrogen can attack the active sites in the carbon matrix and form nitrogen-doped sheets (more porous morphology supports N_2_ adsorption). However, in such a sample, due to the use of molecular nitrogen, the N-doping process is not as effective (N_2_ is a very stable molecule due to the strength of the nitrogen–nitrogen triple bond, so a high amount of energy is required to break the bonds) and a lower amount of nitrogen has been substituted into carbon matrix. In both cases, due to the higher electronegativity of nitrogen, lithium kinetics and storage capabilities are significantly improved [52,53,54]. The favorable changes in local electron density among N atoms causes the drop of the activation energy and allows the capture of more lithium-ions facilitating Li^+^ storage. N-6 species provide more open Li^+^ diffusion channels (active sites) improving fast transportation. N-doping also results a positive impact on the SEI layer formation process (decrease of its formation potential as well as enhanced stability).

## Figures and Tables

**Table 1:** Elemental analysis results of nitrogen-doped and pure carbon aerogels.

Sample	Description of the Sample	Chemical Composition			
		C (wt. %)	H (wt. %)	N (wt. %)	Others (wt. %)
MCAGPS-N8.0% *	MOA_PS ** + 20 wt. % of melamine pyrolysed in Ar	79.02	1.775	7.96	11.245
MCAGPS-N2.3%	MOA_PS + 5 wt. % of melamine pyrolysed in Ar	87.66	1.563	2.28	8.497
MCAGPS-N0.3%	MOA_PS + 1 wt. % of melamine pyrolysed in Ar	89.11	1.869	0.30	8.721
CAGPS-N1.2% ***	OA_PS **** pyrolysed in N_2_	92.55	1.405	1.18	4.865
CAGPS *****	OA_PS pyrolysed in Ar	93.96	1.201	0.04	4.799

* MCAGPS—carbon aerogel made from potato starch modified with melamine; ** MOA_PS—organic aerogel made from potato starch modified with melamine;*** CAGPS-N—carbon aerogel made from potato starch modified by N_2_ pyrolysis; **** OA_PS—organic aerogel made from potato starch; ***** CAGPS—pristine carbon aerogel.

## Data Availability

The data presented in this study are available on request from the corresponding author.
